# The value of ^18^F-FDG-PET/CT in the diagnosis of solitary pulmonary nodules: A meta-analysis

**DOI:** 10.1097/MD.0000000000010130

**Published:** 2018-03-23

**Authors:** Zhen-Zhen Li, Ya-Liang Huang, Hong-Jun Song, You-Juan Wang, Yan Huang

**Affiliations:** a Health Management Center, West China Hospital of Sichuan University; b Department of Nephrology and Rheumatology, Affiliated Hospital/Clinical Medical College of Chengdu University; c Outpatient Department, West China Hospital of Sichuan University, Chengdu, Sichuan, China.

**Keywords:** diagnosis, fluorodeoxyglucose, meta-analysis, PET/CT, solitary pulmonary nodules

## Abstract

**Background:**

Solitary pulmonary nodules (SPNs) are common imaging findings. Many studies have indicated that ^18^F-fluorodeoxyglucose positron emission tomography/computed tomography (^18^F-FDG-PET/CT) is an accurate test for distinguishing benign and malignant SPNs. The aim of this study was to investigate the value of ^18^F-FDG-PET/CT in the diagnosis of malignant SPNs.

**Methods:**

We systematically searched the *PubMed* and *Embase* databases up to March 2017, and published data on sensitivity, specificity, and other measures of diagnostic accuracy of ^18^F-FDG-PET/CT in the diagnosis of malignant SPNs were meta-analyzed. Statistical analyses were undertaken using Meta-DiSc 1.4 software and Stata version 12.0. The measures of accuracy of ^18^F-FDG-PET/CT in the diagnosis of malignant SPNs were pooled using random-effects models.

**Results:**

A total of 20 publications reporting 21 studies were identified. Pooled results indicated that ^18^F-FDG-PET/CT showed a diagnostic sensitivity of 0.89 (95% confidence interval [CI], 0.87–0.91) and a specificity of 0.70 (95% CI, 0.66–0.73). The positive likelihood ratio was 3.33 (95% CI, 2.35–4.71) and the negative likelihood ratio was 0.18 (95% CI, 0.13–0.25). The diagnostic odds ratio was 22.43 (95% CI, 12.55–40.07).

**Conclusions:**

^18^F-FDG-PET/CT showed insufficient sensitivity and specificity for diagnosing malignant SPNs; it cannot replace the “gold standard” pathology by resection or percutaneous biopsy. Larger studies are required for further evaluation.

## 1 Introduction

A solitary pulmonary nodule (SPN) is defined as a round or oval opacity lung lesion <3 cm in diameter and completely surrounded by pulmonary parenchyma, with no associated lymphadenopathy, atelectasis, or pneumonia.^[1–3]^ SPNs are found approximately 0.2% to 2% in chest radiographs and approximately 10% to 40% in computed tomography (CT) scans,^[4–7]^ and cancerous SPN has a reported incidence of 5% to 70%.^[1,8]^ Thus, the early diagnosis of malignant SPNs is key for successful treatment. But the diagnosis of SPNs is a challenge to both clinicians and radiologists, due to the absence of symptoms, nonspecific morphology, and unknown probability of malignancy.^[9]^ For suspicious malignant SPNs, transbronchial needle aspiration biopsy, percutaneous transthoracic biopsy, or video-assisted thoracoscopic surgery can provide “gold standard” histological information, but these are invasive and skill-dependent procedures.^[10–12]^ Recently, ^18^F-fluorodeoxyglucose positron emission tomography/computed tomography (^18^F-FDG-PET/CT), a hybrid imaging combining CT and PET, has proved to be an accurate noninvasive imaging test for distinguishing benign and malignant SPNs.^[2,13,14]^ Some studies have reported that ^18^F-FDG-PET/CT in SPN provides high diagnostic sensitivity (100%) and specificity (96%).^[15]^ Other studies, however, have reported much lower corresponding values of 62% and 25%.^[16,17]^ As the results are inconclusive, we meta-analyzed the available literature to examine the comprehensive status of the diagnostic utility of ^18^F-FDG-PET/CT in SPN. To the authors’ knowledge, this is the first meta-analysis to investigate the diagnostic usefulness of ^18^F-FDG-PET/CT in SPNs.

## 2 Materials and methods

### 2.1 Study selection

Two reviewers searched *PubMed* and *Embase* for meta-analyses related to the diagnostic accuracy of ^18^F-FDG-PET/CT in SPN, but no articles were found. Then, we identified eligible studies up to May 31, 2017. The following search terms were used: “PET/CT” OR “FDG-PET/CT” OR “^18^F-FDG-PET/CT” AND “SPN” OR “solitary pulmonary nodules” OR “lung lesion” AND “sensitivity” AND “specificity” AND “diagnosis.”

The included studies met the following criteria: patients with SPN were from outpatient or inpatient department; ^18^F-FDG-PET/CT imaging was performed in all patients; the number of patients and information about the sensitivity and specificity of 18F-FDG-PET/CT for the diagnosis of SPN was complete; there were clear diagnostic criteria and sizes of the nodule were provided; and the articles were written in English. Exclusion criteria included unpublished data, case reports, letters to the editor, abstracts, and review articles. All analyses were based on previously published studies; thus, no ethical approval and patient consent are required.

### 2.2 Data extraction and quality assessment

Two reviewers independently assessed study eligibility in accordance with the inclusion/exclusion criteria. In cases of disagreement, they consulted for resolution. The standard procedure was performed to extract data from the studies. The following information was collected: first author, country, year of publication, numbers of cases, sensitivity and specificity data, true positives, false positives, true negatives, false negatives, standard uptake value (SUVmax), nodule size, diagnosis standard, and the final histological diagnoses of the nodules. The methodological quality of the studies included in our meta-analysis was assessed using the Quality Assessment of Diagnostic Accuracy Studies (QUADAS-2) checklist, which is more detailed and rigorous than QUADAS, and the maximum score was 11.^[18]^

### 2.3 Statistical analyses

Standard recommended methods were used for the meta-analyses of the diagnostic test evaluations.^[19]^ The data analyses were performed using Meta-DiSc software and STATA version 12.0. The following measures of test accuracy were computed to assess the accuracy of ^18^F-FDG-PET/CT for the diagnosis of SPN: sensitivity, specificity, positive likelihood ratio (PLR), negative likelihood ratio (NLR), and diagnostic odds ratio (DOR). The SROC curves were pooled to evaluate the overall diagnostic performance.^[19,20]^ Random-effect modeling was performed to meta-analyze the diagnostic measures.^[21,22]^ Heterogeneity of studies was evaluated using *I*^2^ and Fisher exact tests. A meta-regression analysis was conducted if significant heterogeneity existed among the included studies, and the following variables were used: SUVmax (2.5 vs −2.5), SPNs diagnosis standard (histology vs histology and follow-up), publication year (before 2010 vs after 2010), and methodological quality (≤9 vs >9). Deeks’ funnel plots were used to test for the potential presence of publication bias.^[23]^

## 3 Results

### 3.1 Study characteristics

The initial literature searches and title review found 52 publications relevant to the search strategy; 27 were excluded based on abstract review. The remaining 25 articles were read in full, and 5 were excluded after the full text was read as they did not display sufficient data on nodule size.^[24–28]^ Thus, 20 publications assessing the diagnostic performance of ^18^F-FDG-PET/CT in SPNs were included.^[15–17,29–45]^ One study involved 2 study groups ^[39]^; sufficient data were reported for each that they were treated as 2 independent studies. Finally, 21 studies from 20 publications were included. Characteristics of the selected studies are summarized in Table 1 . There were 1557 patients with SPNs, 918 malignant and 639 benign. SPNs were diagnosed based on histology in 12 studies^[15–17,31,33,35,36,39,40,43–45]^; in the remaining 9 studies, diagnosis was based on histology or follow-up. All the SPNs were <3 cm. The SUVmax was not exactly the same.

**Table 1:**
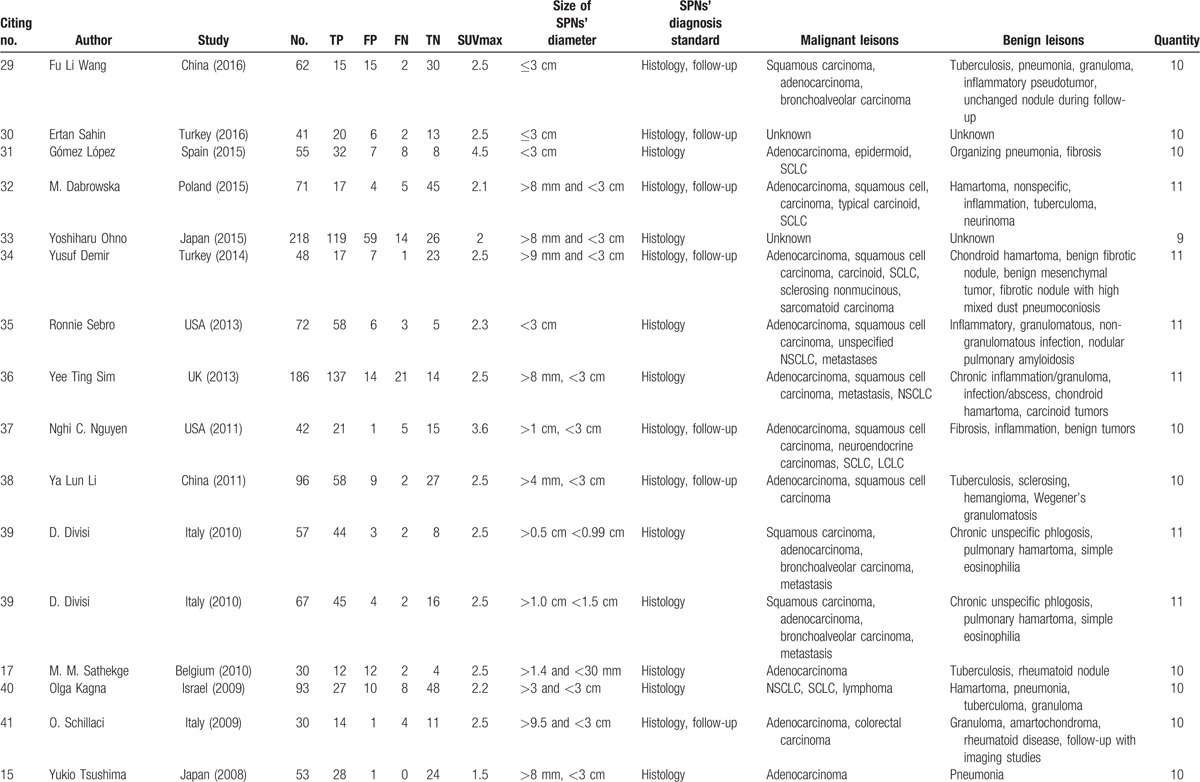
Characteristics of studies included in the meta-analysis^a^.

**Table 1 (Continued):**
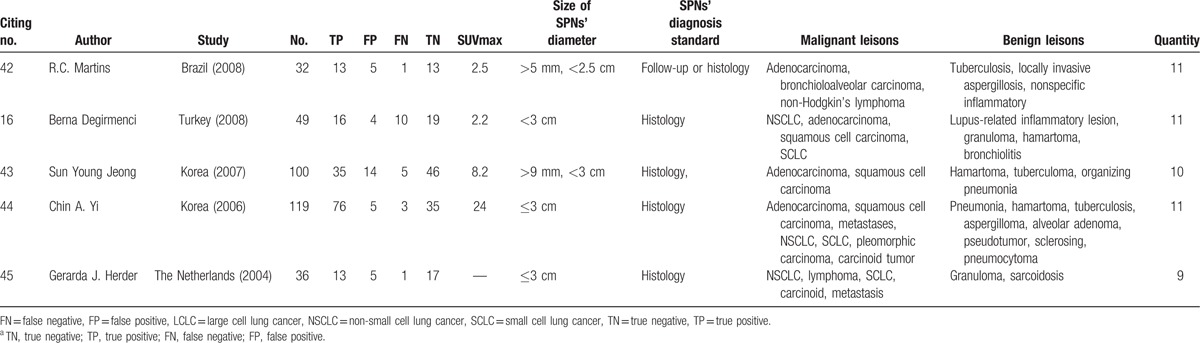
Characteristics of studies included in the meta-analysis^a^.

### 3.2 Meta-analysis results

**Figure 1:**
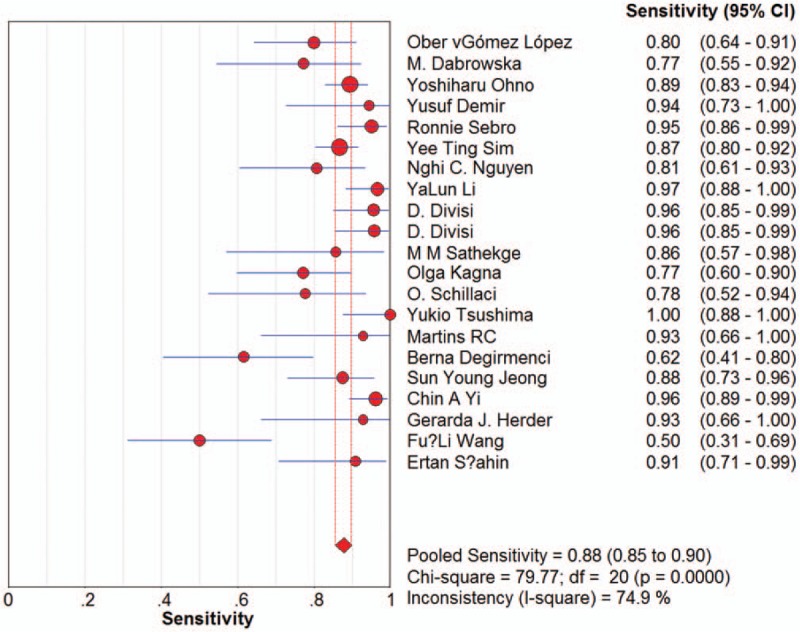
Forest plot of estimates of sensitivity for ^18^F-FDG-PET/CT in the diagnosis of malignant SPNs. Point estimates of sensitivity from each study are shown as solid circles, the size of which reflects the total number of cases and controls. Error bars show 95% confidence intervals. Numbers indicate the reference numbers of the studies.

**Figure 2:**
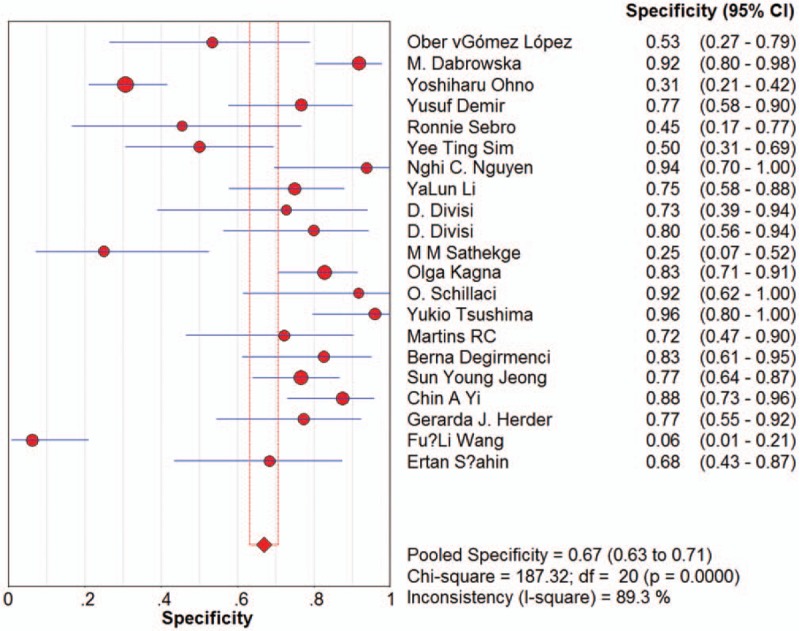
Forest plot of estimates of specificity for ^18^F-FDG-PET/CT in the diagnosis of malignant SPNs. Point estimates of specificity from each study are shown as solid circles, the size of which reflects the total number of cases and controls. Error bars show 95% confidence intervals. Numbers indicate the reference numbers of the studies.

**Figure 3:**
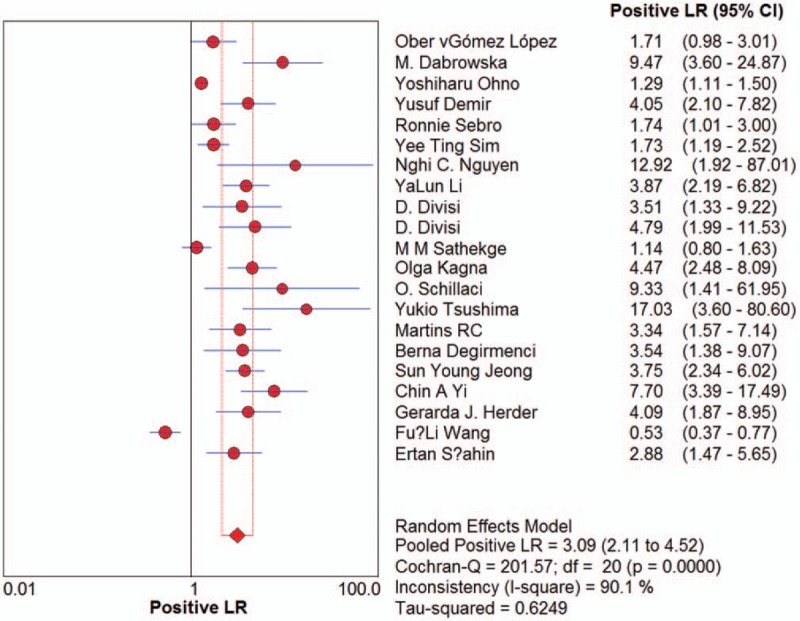
Forest plot of estimates of positive likelihood ratios for ^18^F-FDG-PET/CT in the diagnosis of malignant SPNs. Point estimates of the positive likelihood ratios from each study are shown as solid circles, the size of which reflects the total number of cases and controls. Error bars show 95% confidence intervals. Numbers indicate the reference numbers of studies.

**Figure 4:**
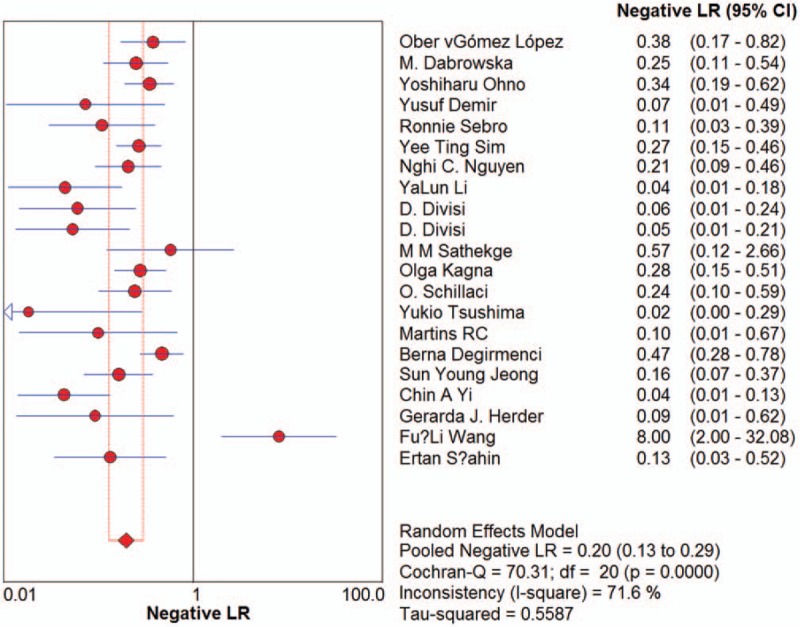
Forest plot of estimates of the negative likelihood ratios for ^18^F-FDG-PET/CT in the diagnosis of malignant SPNs. Point estimates of negative likelihood ratios from each study are shown as solid circles, the size of which reflects the total number of cases and controls. Error bars show 95% confidence intervals. Numbers indicate the reference numbers of the studies.

**Figure 5:**
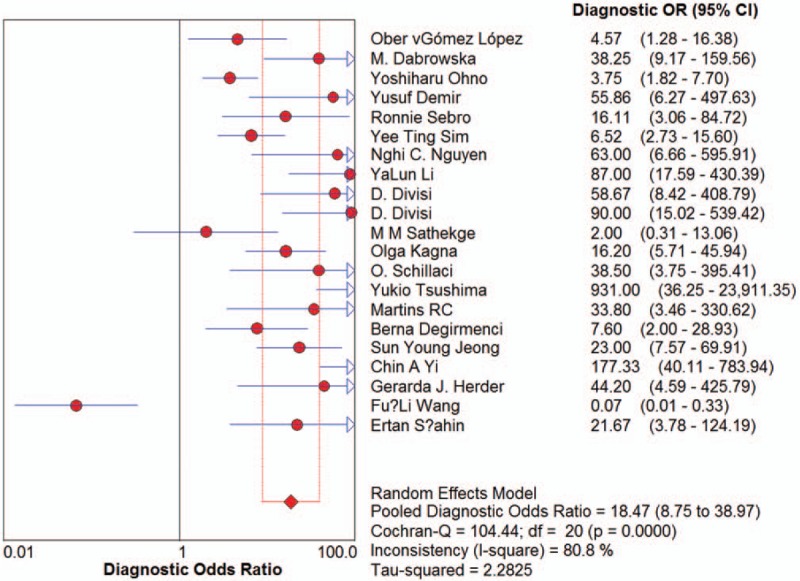
Forest plot of estimates of diagnostic odds ratios for ^18^F-FDG-PET/CT in the diagnosis of malignant SPNs. Point estimates of diagnostic odds ratios from each study are shown as solid circles, the size of which reflects the total number of cases and controls. Error bars show 95% confidence intervals. Numbers indicate the reference numbers of the studies.

Sensitivity for ^18^F-FDG-PET/CT in SPN diagnosis ranged from 0.62 to 1 in the 21 studies, and meta-analysis of sensitivity indicated a pooled sensitivity of 0.89 (95% confidence interval [CI], 0.87–0.91) (Fig. 1). Specificity ranged from 0.25 to 0.96, and pooled specificity was 0.70 (95% CI, 0.66–0.73) (Fig. 2). The PLR was 3.33 (95% CI, 2.35–4.71) (Fig. 3) and the NLR was 0.18 (95% CI, 0.13–0.25) (Fig. 4). The DOR was 22.42 (95% CI, 12.55–40.07) (Fig. 5).

**Figure 6:**
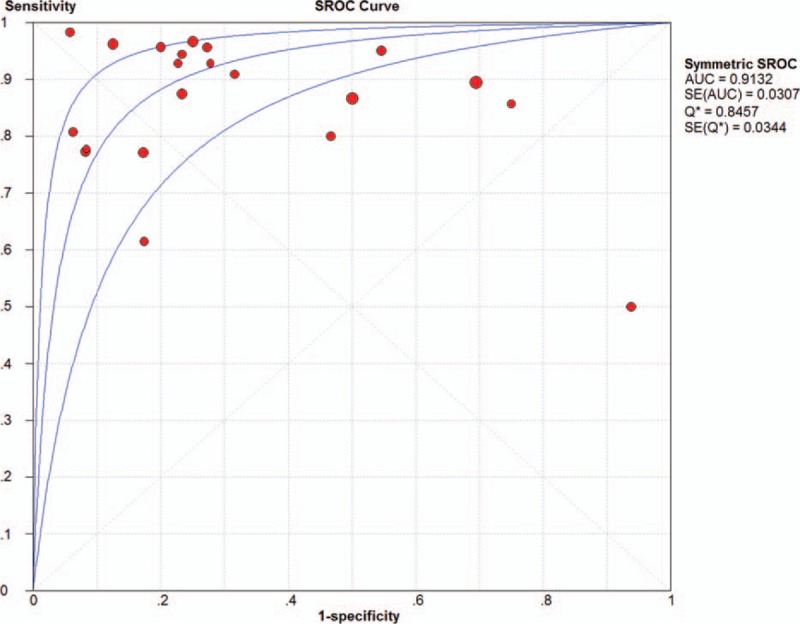
Summary receiver operating characteristic curves for ^18^F-FDG-PET/CT. Each study is depicted as a solid circle, the size of which reflects the total number of cases and controls.

Summary receiver-operating characteristic (SROC) curves were generated by plotting sensitivity against 1 specificity for individual studies. The maximum joint sensitivity and specificity was 0.89, with an area under the curve (AUC) of 0.9084 (SEM 0.0254) (Fig. 6).

### 3.3 Meta-regression analysis

*I*^2^ values for the pooled diagnostic performance parameters were high: 62.3% for sensitivity, 84.8% for specificity, 87.4% for PLR, 60.0% for NLR, and 67.4% for DOR (all *P* < .05), indicating significant heterogeneity. To identify possible reasons for this heterogeneity, we conducted a meta-regression to assess the effect of the study quality on the relative DOR (RDOR) of ^18^F-FDG-PET/CT for SPNs. The characteristics of the covariates are shown in Table 1 . Diagnostic accuracy was not significantly affected by the SUVmax value (*P* = .3399), SPN diagnosis standard (*P* = .2605), methodological quality (*P* = .3578), or publication year (*P* = .3707). The meta-regression results are shown in detail in Table 2.

**Table 2:**
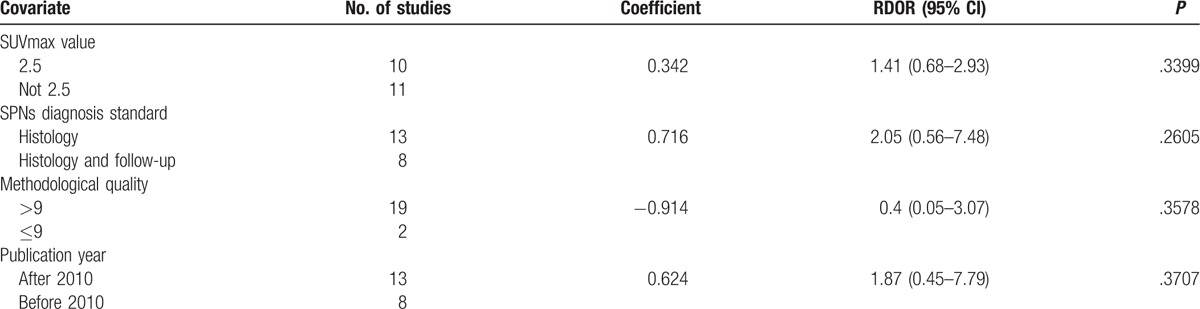
Meta-regression of the diagnostic accuracy of ^18^F-FDG-PET/CT.

### 3.4 Publication bias

**Figure 7:**
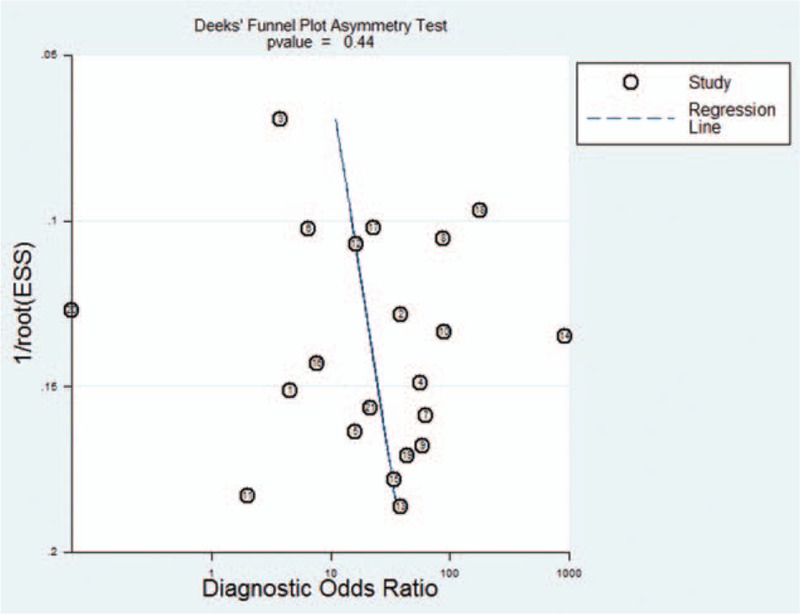
Funnel plot for evaluating publication bias among the 18 studies included in the meta-analysis. The log of the DOR is plotted against the standard error of the log DOR; the latter serves as an indicator of the sample size. Each article is shown as a solid circle, and the regression line is shown.

Deeks test gave a *P* value of .26, suggesting that our analysis had no significant risk of publication bias (Fig. 7).

## 4 Discussion

SPNs are a common clinical problem. The histologies of SPNs can be benign tumors, infectious lesions, and lung cancer. The prevalence of lung cancer in SPNs is high, and the early identification of malignant nodules can help improve the chance of successful treatment. Transbronchial needle aspiration biopsy, percutaneous transthoracic biopsy, or video-assisted thoracoscopic surgery can provide histological information. But because these are invasive procedures depending on nodule diameter and position, and are skill-dependent, they have variable accuracy in lung cancer diagnosis. PET/CT with ^18^F-FDG is widely used in the diagnosis of SPNs. ^18^F-FDG-PET/CT can detect the presence of metabolically active tissue by quantifying the rate of cell glucose metabolism. Malignant nodules always have increased expression of the glucose transporter and elevated metabolic activity evidenced by a high FDG uptake.^[46]^ But sometimes benign lesions also have increased metabolic activity such as infections, tuberculosis, and granulomatous disease.^[1,47,48]^

Our analysis suggests that ^18^F-FDG-PET/CT measurements alone are not sufficiently sensitive (0.89) and specific (0.70) to diagnose SPNs. Meta-analysis of the 21 included studies indicated a pooled DOR of 22.43 for ^18^F-FDG-PET/CT. The DOR is the ratio of the odds of ^18^F-FDG-PET/CT being positive in malignant SPNs relative to the odds of it being positive in benign SPNs. The SROC curve and the area underneath it present a trade-off between sensitivity and specificity.^[49]^ Meta-analysis showed that the area under the SROC curve was 0.9084. These results indicate a relatively high accuracy. DOR and SROC curve analyses are difficult to interpret and use in clinical practice,^[50]^ as the likelihood ratios are more clinically meaningful for measuring diagnostic accuracy.^[50,51]^ The PLR value of 3.33 suggests that malignant SPNs have approximately 3-fold higher chance of being ^18^F-FDG-PET/CT positive compared with benign SPNs; this is insufficient to serve as the sole basis for diagnosing malignant SPNs. Concomitantly, the NLR was 0.18, meaning it had an 18% probability that the SPNs were malignant if the ^18^F-FDG-PET/CT was negative. There were both false positives and false negatives. The increased false positives were due to findings in areas with a high prevalence of tuberculosis and granulomatous disease, while false negatives occurred in small nodules, hyperglycemia, and tumors with low metabolic activity.^[52]^ This also indicates that such a measurement is inadequate for ruling out malignant nodules on its own.

Heterogeneity among the included studies determined the reliability of meta-analyses; we found significant heterogeneity among the studies of our meta-analysis, so the results should be interpreted with caution. We checked the 21 studies carefully to find the possible factors that caused this heterogeneity. The analysis suggested SUVmax value differences and SPN diagnosis standards. The definite nature of the nodule was determined on the histologic findings and/or radiological follow-up. Concomitantly, the methodological quality and publication years varied among the included studies, but the meta-regression suggested that they did not affect the diagnostic performance of ^18^F-FDG-PET/CT. Therefore, the basis for the heterogeneity in our meta-analysis is unclear.

The pathologic analysis classified SPNs as malignant or benign. Among the malignant SPNs, the most prevalent histologies were adenocarcinoma, squamous cell carcinoma, and small cell lung cancer. Other histologies were found, such as lymphoma, non-Hodgkin's lymphoma, and epidermoid. In the benign SPNs, the most prevalent histologies were tuberculosis, pneumonia, and granuloma. The differences in histologies were significant, and they had obviously different ^18^F-fluorodeoxyglucose uptake. The studies varied in subject age and in stages of malignant SPNs. Benign SPNs in the control groups also were not at the same stage. These factors may have affected the diagnostic accuracy.

The meta-analysis had several limitations. First, only studies identified in a few databases were included, possibly leading to the exclusion of high-quality non-English research. Second, the SUVmax and size of the nodules in the studies were not exactly the same, leading to increased heterogeneity. Third, the pathological diagnoses of malignant nodules in the 19 studies did not the same type, for example, some malignant nodules were squamous carcinoma, some were adenocarcinoma or bronchoalveolar carcinoma, those would possibly leading to significant heterogeneity. In addition, other important factors contributed to the pooled result, such as past history and environmental exposure; these issues could not be precisely explained due to insufficient information.

Although PET-CT is and will continue to be a valuable noninvasive imaging tool for the diagnosis of SPNs, there remain many pitfalls in false-positive and false-negative lesions; it cannot replace the “gold standard” pathology of resection or percutaneous biopsy.

## 5 Author contributions

**Conceptualization:** Y.-L. Huang, Y. Huang, Y. Wang.

**Data curation:** Z.-Z. Li, Y.-L. Huang, H. Song.

**Formal analysis:** Z.-Z. Li, H. Song.

**Funding acquisition:** Y. Wang.

**Investigation:** Z.-Z. Li, Y. Huang.

**Methodology:** Z.-Z. Li, Y.-L. Huang, H. Song.

**Project administration:** Y. Huang, Y. Wang.

**Resources:** H. Song.

**Software:** H. Song.

**Supervision:** Y. Huang, Y. Wang.

**Validation:** Y. Wang.

**Visualization:** Y. Wang.

**Writing – original draft:** Z.-Z. Li.

**Writing – review & editing:** Y. Huang, Y. Wang.
